# Annotations

**Published:** 1902-08-30

**Authors:** 


### Annotations.

### Science and the Horse Dealer.

In his annual report, Mr. A. Spencer, chief officer of the Public Control Department of the London County Council, gives the number of cases of glanders which were reported during the year as 1,857, many of which were detected solely by aid of mallein, which had been injected on account of the animals having been in contact with diseased horses. Mallein has also been used in many other cases as an aid to diagnosis when the external evidence of the disease was slight or not sufficient to enable the veterinary inspectors to condemn the animals as diseased. Indeed the diagnostic utility of mallein has been well marked, and Mr. Spencer astutely suggests that the increased prevalence of glanders may be due to unscrupulous owners of horses having made use of mallein for their own ends. As he says "mallein is useful to unscrupulous owners in enabling them quietly to weed out suspicious cases from their studs," quite ignoring what will happen in the stable of the purchaser. To such vile uses does the ingenious horse dealer turn the discoveries of science. Hence, as Mr. Spencer further tells us, it "becomes more than ever important for owners to test new purchases by injecting them with mallein before permitting them to come into contact with existing studs." Evidently we must set a microbe to catch a microbe.

### "Bracing" or "Relaxing."

There are few subjects on which people are more ready to express a positive opinion than in regard to the "bracing" or "relaxing" air of certain places. Yet when we inquire what our good friends mean by the terms they use so glibly the result is far from satisfactory. "Bracing? a place that makes you feel 'fit' you know." "Relaxing? anything that makes one 'slack' and takes the 'go' out of one." We get no further, and in truth the answer to the question is somewhat complex. Putting on one side those electrical conditions which in almost any place may render some people headachy and depressed, the problem is chiefly one of moisture of air, movement of air, and the relation of air-heat and sun-heat. Roughly it may be said that the more nearly the air (for its temperature) is saturated with moisture, the stiller it is, and the more the sense of warmth depends upon warmth in the air itself, the more relaxing will a climate be found. Comparative dryness and coolness of air are essential factors in the making of a bracing climate. Even in Egypt the air is proportionately cool except in the hot winds, and then what place is more miserable? Hence the presence of woods, or of profuse vegetation of any kind, the existence of ground water near the surface, the neighbourhood of rivers and marshes, and in general flatness of land tends to those conditions termed relaxing. On the other hand, mountains and hill sides, but not deep mountain valleys; the sparsely turfed chalk downs, but not chalk "bottoms"; open seaside places, but not "under-cliff" bays protected from the wind, are often bracing places. It must be remembered that bracing as the seaside is often felt to be by town visitors, it is so in spite of its moisture. It is the purity, the movement, and the comparative coolness of sea air that makes it bracing. Stagnant air is as relaxing at the seaside as anywhere else, and, indeed, more so than at most places. The most bracing places of all are to be found on the sides of hills in mountain districts. Of all sorts of air, that which is both moist and warm, and especially that which remains warm at night is the most relaxing. One great advantage of living on the slope of a hill-side is that the air in such a situation is never still at night, while one of the evils of the plains is that except when moved by outside winds the air lies stagnant the night through. From all of which it will be gathered that, other things being equal, it is on the prevalence of certain kinds of weather rather than geological conditions or geographical position that goes to making of a bracing climate.

### Physical Training and Free Meals.

It has long been held by many that the compulsory education of the starveling is as absurd as it is cruel. Indeed, if one regards the whole problem from a physiological point of view, compulsory education becomes a desperately big business involving some wide excursions into socialism. It surely is sublimely illogical to force a starving child to go to school and yet to refuse to give it that food without which all the expensive education lavished upon it must lead to nothing. Look at all we spend upon school buildings, upon teachers, upon educational machinery, and then look upon the dull and drowsy child for whose benefit we expend it—dull and drowsy because its stomach is empty and its brain is starved. Yet after scattering money with so free a hand upon the teaching we draw back from giving the odd pennies for the breakfast; for that spells socialism. Perhaps we are right, but at least we are illogical. If, however, as seems to be foreshadowed by the appointment of the Royal Commission on Physical Training (Scotland), any experiment should be made in the direction of introducing physical training (which involves muscular work) into our elementary schools (which are compulsory), the matter of meals would become a very urgent one. Several of the witnesses, including Sir Lauder Brunton, were asked by the Commissioners to give an opinion upon this question of food. If extra physical exercises were to be given should extra food be insisted upon? and if so who was to supply it? Sir Lauder Brunton appeared to think that if the exercises created increased appetite the parents would provide. But we doubt whether the question can be regarded as settled by so simple a solution. It is not a question of appetite, but of using up the child's energy. If ordinary people have not so far "enthused" over free breakfast schemes, this has to some extent been due to their confidence in the empty and tired child dropping to sleep and so "not being much the worse" for going to school. But even the man in the street can see that "physical training" will "take it out of a child," and he will say that those who take it out must put it in. Any large extension of physical training in elementary schools will raise many very pretty little questions in practical socialism.

